# CORRELATIONS AMONG SLEEP BIOMETRICS, DAILY STEPS, AND SELF-REPORTED FUNCTION IN OLDER COUPLES WITH AND WITHOUT MCI

**DOI:** 10.1093/geroni/igae098.3311

**Published:** 2024-12-31

**Authors:** Kourosh Alimohammadbeik, Cynthia Jacelon

**Affiliations:** University of Massachusetts Amherst, Amherst, Massachusetts, United States; University of Massachusetts Amherst, Amherst, Massachusetts, United States

## Abstract

Altered sleep patterns are common among older individuals and those with Mild Cognitive Impairment (MCI). Sleep changes can lead to fatigue, and alterations in physical and social function. In couples, disrupted sleep of one partner can affect the other’s sleep patterns. We compared measures of sleep and steps, captured by actigraph, with self-reported fatigue, physical function, and social roles among cognitively intact couples, and those with one partner who had MCI. The WHO International Classification of Functioning, Disability, and Health was the framework. Using a correlational design, we compared biometric measures with scales on the PROMIS 29 at three points in time in 13 couples >70 years old. Eight couples had one partner who scored < 25 on the MoCA. Significance was set at P =0.05. In cognitively intact couples, total and deep sleep time were correlated with fatigue (negative) and social roles. Deep sleep time was associated with physical function. Bedtime and time awake were correlated with fatigue (negative) and social roles. In couples with MCI, total and deep sleep time were associated with fatigue (negative). Steps correlated with physical activity, fatigue (negative), and social roles. Time awake was positively associated with fatigue. Bedtime and number of awakenings were associated with social role (negative). Sleep quality and steps have significant effects on self-reported function of couples with and without MCI. Total and deep sleep time were most important for cognitively intact couples, and number of steps was most important for couples with MCI.

